# Supplementary material for: Enhanced fish production during a period of extreme global warmth
Source: Nat Commun. 2020 Nov 6;11:5636. doi: 10.1038/s41467-020-19462-w (PMC7648762; doi:10.1038/s41467-020-19462-w)
Supplement: Supplementary file 1 — Supplementary Information [file 41467_2020_19462_MOESM1_ESM.pdf]

Supplementary Information:

Enhanced fish production during a period of extreme global warmth

Gregory L. Britten, Elizabeth C. Sibert

Supplementary Figures

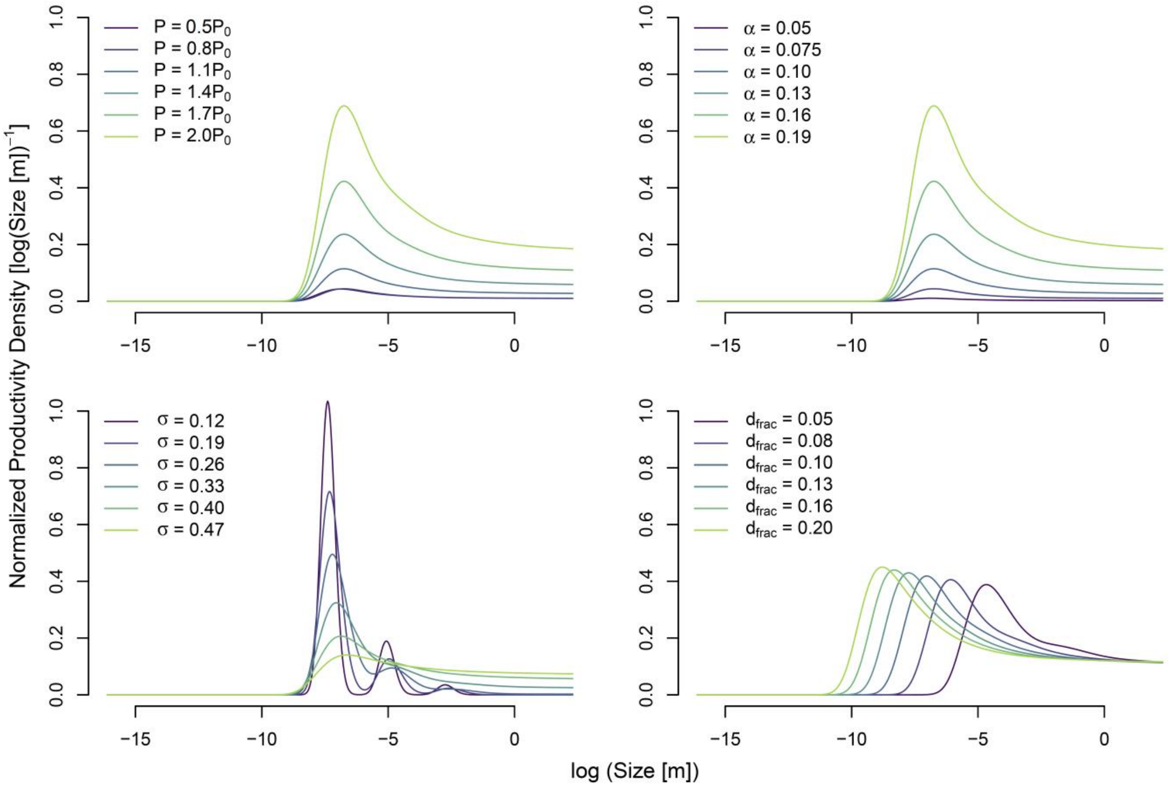

Supplementary Figure 1. Parameter sensitivity analyses demonstrating the effects of each parameter. Plots were produced by holding all but one parameter at their reference values. The varying parameter value is given in the legend. Parameter P is the primary production scale factor, α is the trophic transfer efficiency, σ is the standard deviation of the prey size distribution, and d_{frac} is the mean of the prey size distribution expressed as a fraction of the predator size.

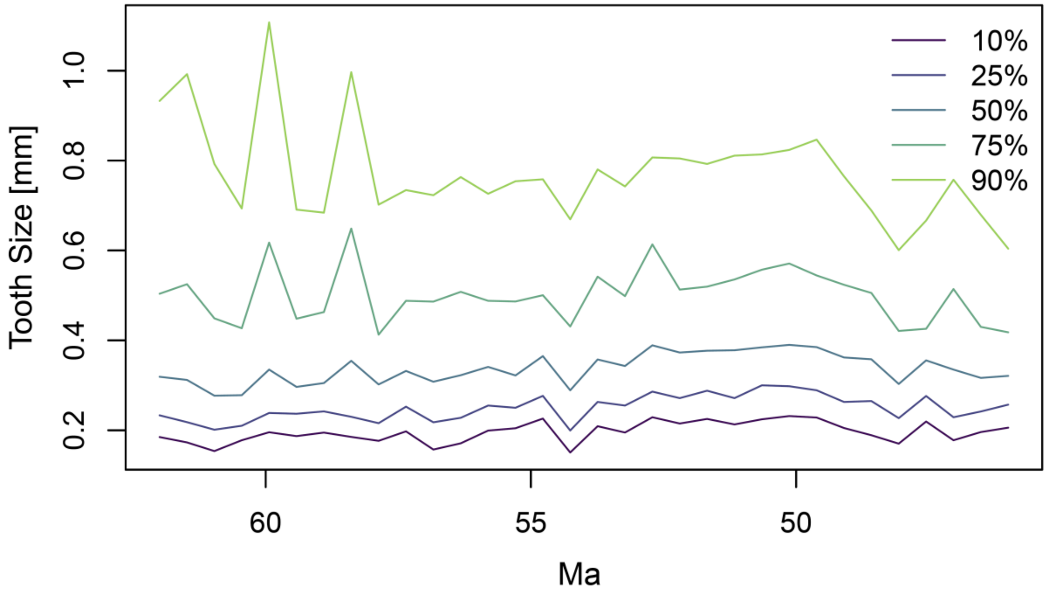

Supplementary Figure 2. Quantile time series of the observed ichthyolith size distribution over time. Each plot represents a different quantile of the size distributions plotted over time.

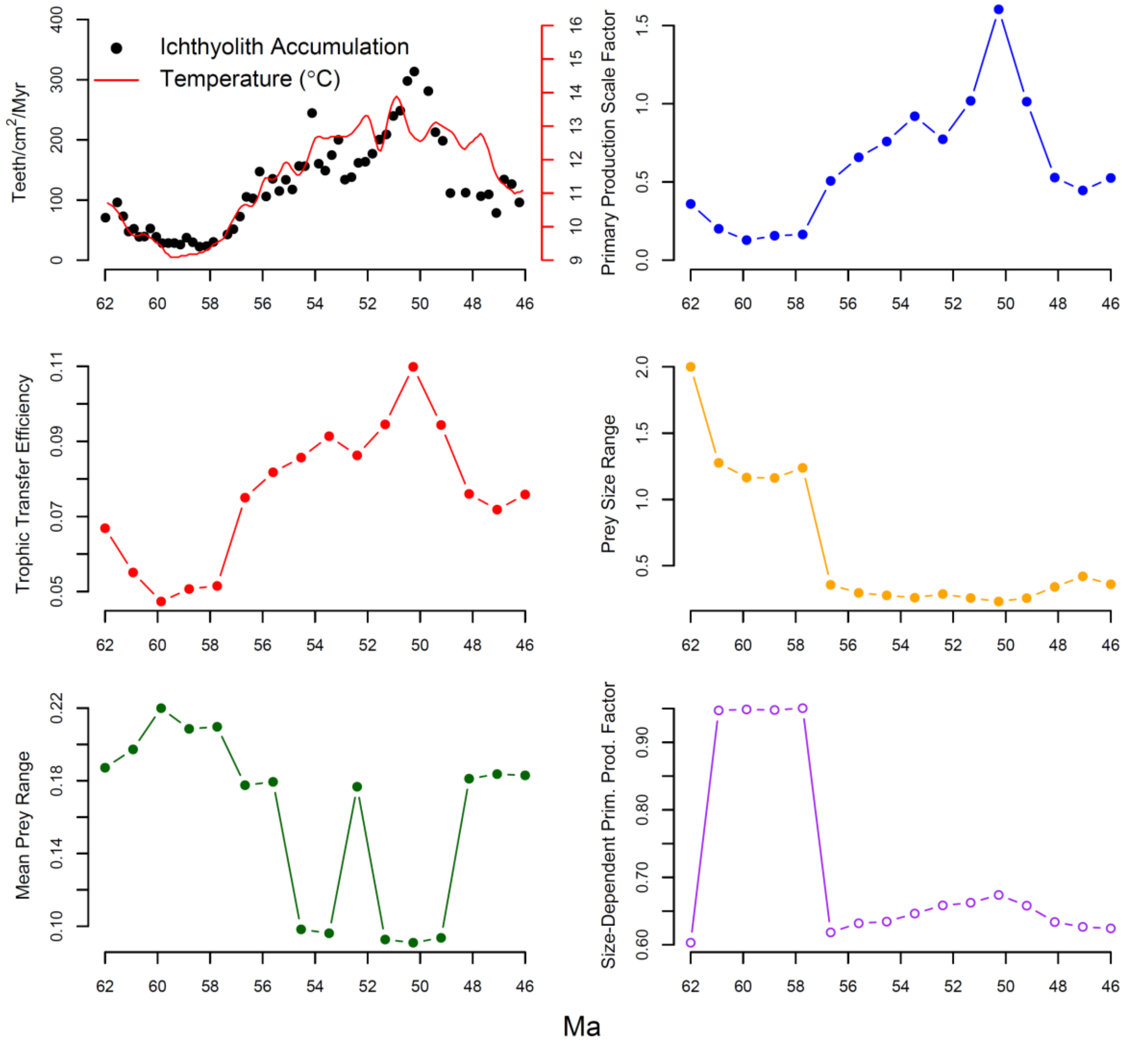

Supplementary Figure 3. Parameter time series from the time-varying analysis. The top left panel repeats Figure 1a from the main text for context. All other figures show the optimized time-varying parameter according to 1-Myr time bins.
