# Supplementary material for: Enhanced fish production during a period of extreme global warmth
Source: Nat Commun. 2020 Nov 6;11:5636. doi: 10.1038/s41467-020-19462-w (PMC7648762; doi:10.1038/s41467-020-19462-w)
Supplement: Supplementary file 3 — Reporting Summary [file 41467_2020_19462_MOESM3_ESM.pdf]

nature research

Corresponding author(s): Gregory L. Britten (gbritten@mit.edu)

Last updated by author(s): 10/13/2020

Reporting Summary

Nature Research wishes to improve the reproducibility of the work that we publish. This form provides structure for consistency and transparency in reporting. For further information on Nature Research policies, see Authors & Referees and the Editorial Policy Checklist.

Please do not complete any field with "not applicable" or n/a. Refer to the help text for what text to use if an item is not relevant to your study.
For final submission: please carefully check your responses for accuracy; you will not be able to make changes later.

Statistics

For all statistical analyses, confirm that the following items are present in the figure legend, table legend, main text, or Methods section.

n/a	Confirmed	
☐	☒	The exact sample size (n) for each experimental group/condition, given as a discrete number and unit of measurement
☐	☒	A statement on whether measurements were taken from distinct samples or whether the same sample was measured repeatedly
☐	☒	The statistical test(s) used AND whether they are one- or two-sided<br>*Only common tests should be described solely by name; describe more complex techniques in the Methods section.*
☐	☒	A description of all covariates tested
☐	☒	A description of any assumptions or corrections, such as tests of normality and adjustment for multiple comparisons
☐	☒	A full description of the statistical parameters including central tendency (e.g. means) or other basic estimates (e.g. regression coefficient) AND variation (e.g. standard deviation) or associated estimates of uncertainty (e.g. confidence intervals)
☒	☐	For null hypothesis testing, the test statistic (e.g. F, t, r) with confidence intervals, effect sizes, degrees of freedom and P value noted<br>*Give P values as exact values whenever suitable.*
☒	☐	For Bayesian analysis, information on the choice of priors and Markov chain Monte Carlo settings
☒	☐	For hierarchical and complex designs, identification of the appropriate level for tests and full reporting of outcomes
☒	☐	Estimates of effect sizes (e.g. Cohen's d, Pearson's r), indicating how they were calculated

Our web collection on statistics for biologists contains articles on many of the points above.

Software and code

Policy information about availability of computer code

Data collection	ImageJ 1.48 was used to measure the ichthyoliths included in this study
Data analysis	R version 3.6.1; analysis code is available at https://doi.org/10.5281/zenodo.4095198

For manuscripts utilizing custom algorithms or software that are central to the research but not yet described in published literature, software must be made available to editors/reviewers. We strongly encourage code deposition in a community repository (e.g. GitHub). See the Nature Research guidelines for submitting code & software for further information.

Data

Policy information about availability of data

All manuscripts must include a data availability statement. This statement should provide the following information, where applicable:

- Accession codes, unique identifiers, or web links for publicly available datasets
- A list of figures that have associated raw data
- A description of any restrictions on data availability

The ichthyolith accumulation data analyzed in this study are available at https://doi.pangaea.de/10.1594/PANGAEA.846789. The individual ichthyolith size measurements are also downloadable from https://doi.org/10.5281/zenodo.4095198. The images analyzed in this study are available at https://doi.org/10.5061/dryad.prr4xgxj4

Field-specific reporting

Please select the one below that is the best fit for your research. If you are not sure, read the appropriate sections before making your selection.

☐ Life sciences ☐ Behavioural & social sciences ☒ Ecological, evolutionary & environmental sciences

For a reference copy of the document with all sections, see nature.com/documents/nr-reporting-summary-flat.pdf

Ecological, evolutionary & environmental sciences study design

All studies must disclose on these points even when the disclosure is negative.

Study description	We analyze a time series of fossil properties by fitting a mechanistic ecological model to the data. This required use of software to minimize a cost function that describes the quantitative misfit between the model predictions and observations.
Research sample	A "sample" is all of the microfossil fish teeth that are retained on a 106 micon sieve, from a discrete 5-15 gram sample of deep-sea sediment. The "samples" are taken at regular intervals throughout a sediment core. This is a standard measurement for micropaleontological studies, and the number of teeth per gram is converted to an accumulation rate. The size threshold was chosen to get a sufficiently large number of ichthyoliths to provide a statistically significant sample (>50 fossils per discrete sediment sample).
Sampling strategy	Sample size is limited by the diameter of deep-sea cores, which are small and extremely expensive to obtain - it is impossible to collect replicate cores, so samples are processed in a random order to reduce the possibility of bias and evaluate variability.
Data collection	Sediment samples are disaggregated and washed using DI water, and all fossils are picked out and archived on microfossil slides. This was all done by one person (author E. C. S.). The slides were then imaged using a Canon Powershot S5 IS microscope-mounted camera. The sizes of each individual tooth were measured using ImageJ, and each image was size-calibrated using an occular micrometer, also by E.C.S.
Timing and spatial scale	The original sediment core was collected in 1987 by the Deep Sea Drilling Project, and stored in the IODP Gulf Coast Repository. The individual samples were requested and processed by ECS during an 8-month period in 2013 and 2014. As the samples are not of living organisms, the timing and nature of sampling does not have any influence on the results, however it is worth noting that as the samples were all processed in a short period of time, researcher bias and experience in finding and quantifying the teeth did not vary.
Data exclusions	Although there is size data for ichthyolith assemblages beyond the period of study, the time interval included (62-46 million years ago) was chosen, because it excludes the Cretaceous-Paleogene Mass Extinction and rapid evolution and restructuring of fish communities associated with it (for samples >62 Ma), which we felt would introduce additional biases. Further, samples <46 Ma were excluded as after this time, the ice volume building up on Antarctica confounds global temperature estimates, thus reducing our confidence in the climatic signal. This time interval was chosen prior to beginning the analyses.
Reproducibility	Although it is impossible to replicate deep-sea cores due to the challenging and expensive nature of ocean drilling, the samples were processed in a random (and unknown) order, and assessed for variability.
Randomization	Randomization was not applicable to the predefined spatial sampling design.
Blinding	This study did not include a "control" and a "treatment" group, as it is an observational time-series dataset. Therefore no blinding was necessary in this study. Samples were processed in a way to remove potential time-series biases.

Did the study involve field work? ☐ Yes ☒ No

Reporting for specific materials, systems and methods

We require information from authors about some types of materials, experimental systems and methods used in many studies. Here, indicate whether each material, system or method listed is relevant to your study. If you are not sure if a list item applies to your research, read the appropriate section before selecting a response.

Materials & experimental systems

n/a	Involved in the study	
☒	☐	Antibodies
☒	☐	Eukaryotic cell lines
☐	☒	Palaeontology
☒	☐	Animals and other organisms
☒	☐	Human research participants
☒	☐	Clinical data

Methods

n/a	Involved in the study	
☒	☐	ChIP-seq
☒	☐	Flow cytometry
☒	☐	MRI-based neuroimaging

Palaeontology

Specimen provenance	Specimens were isolated from Deep Sea Drilling Project Site 596, a red clay sediment core from the middle of the South Pacific Ocean, (23°51.20'S, 165°39.27'W) collected in 1987 by the Deep Sea Drilling Project (now the International Ocean Discovery Program). No permits were necessary for this collection as the samples were collected in international waters. The sediment cores have been curated by the IODP since their collection in 1987.
Specimen deposition	IODP cores are available to any person who makes a reasonable request (as determined by the individual curator). The physical microfossil teeth were imaged at high resolution and those images are stored on the DRYAD data repository at https://doi.org/10.5061/dryad.prr4xgxj4
Dating methods	Samples were dated using an established age model for the sediment core, published by Zhou & Kyte 1992 and cited in the manuscript.

☒ Tick this box to confirm that the raw and calibrated dates are available in the paper or in Supplementary Information.

This checklist template is licensed under a Creative Commons Attribution 4.0 International License, which permits use, sharing, adaptation, distribution and reproduction in any medium or format, as long as you give appropriate credit to the original author(s) and the source, provide a link to the Creative Commons license, and indicate if changes were made. The images or other third party material in this article are included in the article's Creative Commons license, unless indicated otherwise in a credit line to the material. If material is not included in the article's Creative Commons license and your intended use is not permitted by statutory regulation or exceeds the permitted use, you will need to obtain permission directly from the copyright holder. To view a copy of this license, visit http://creativecommons.org/licenses/by/4.0/
